# Differences in neuroanatomy and functional connectivity between motor subtypes of Parkinson’s disease

**DOI:** 10.3389/fnins.2022.905709

**Published:** 2022-07-22

**Authors:** Jin Hua Zheng, Wen Hua Sun, Jian Jun Ma, Zhi Dong Wang, Qing Qing Chang, Lin Rui Dong, Xiao Xue Shi, Ming Jian Li, Qi Gu, Si Yuan Chen, Dong Sheng Li

**Affiliations:** ^1^Department of Neurology, Henan Provincial People’s Hospital, Zhengzhou, China; ^2^Department of Neurology, People’s Hospital of Zhengzhou University, Zhengzhou, China; ^3^Department of Neurology, People’s Hospital of Henan University, Zhengzhou, China

**Keywords:** Parkinson’s disease, MRI, voxel-based morphometry, tremor-dominant, postural instability

## Abstract

**Background:**

The “postural instability/gait difficulty” (PIGD) and “tremor-dominant” (TD) motor subtypes of Parkinson’s disease (PD) differ in their clinical manifestations. The neurological basis of these differences is unclear.

**Methods:**

We performed voxel-based morphometric analysis and measured amplitudes of low-frequency fluctuation (ALFF) on 87 PIGD patients and 51 TD patients. We complemented this neuroanatomical comparison with seed-to-voxel analysis to explore differences in functional connectivity.

**Results:**

The PIGD group showed significantly smaller gray matter volume in the medial frontal gyrus (mainly on the right side) than the TD group. Across all patients, gray matter volume in the medial frontal gyrus correlated negatively with severity of PIGD symptoms after controlling for age (*r* = −0.250, *p* = 0.003), but this correlation was not observed in separate analyses of only PIGD or TD patients. The PIGD group showed greater functional connectivity of the right superior frontal gyrus with the left lingual gyrus, right lateral occipital cortex, and right lingual gyrus. ALFF did not differ significantly between the two groups.

**Conclusion:**

Postural instability/gait difficulty may be associated with smaller gray matter volume in medial frontal gyrus than TD, as well as with greater functional connectivity between the right superior frontal gyrus and occipital cortex. These results may help explain the clinical differences between the two motor subtypes of PD.

## Introduction

Parkinson’s disease (PD) is a progressive neurodegenerative disorder whose main clinical manifestations include bradykinesia, tremor, rigidity, and gait/postural disturbance. PD can be further divided into various motor subtypes: tremor-dominant (TD), postural instability/gait difficulty (PIGD), and mixed subtypes (Jankovic et al., 1990). The different subtypes show different clinical course and prognosis (Marras et al., 2002; Rajput et al., 2009). PIGD patients, for example, suffer more rapid motor progression than TD patients, they are at higher risk of dementia and they tend to respond less to medication (Jankovic et al., 1990; Rajput et al., 2009). Exploring the differences between PIGD and TD subtypes may help clarify their neural mechanisms and guide the development of future treatments.

Studies based on voxel-based morphometry (VBM), a classic automated technique in magnetic resonance imaging (MRI) (Ashburner and Friston, 2000), have provided the first clues to neuroanatomical differences between PIGD and TD patients, yet the results have been inconsistent. For example, some researchers have reported smaller gray matter volume (GMV) in all major brain lobes and subcortical areas in PIGD than in TD (Rosenberg-Katz et al., 2013), while others found smaller GMV only in the frontal cortex, and even this lost statistical significance after correcting for covariates (Al-Bachari et al., 2017). Another study found no significant volume differences in any brain region between patients with the akinetic/rigidity subtype of PD and patients with the TD subtype (Karunanayaka et al., 2016). This conflicting literature highlights the need for further neuroanatomical comparisons between PIGD and TD, especially involving groups larger than the 15–30 patients per arm in the abovementioned studies.

Analysis of the amplitudes of low-frequency fluctuations (ALFF) can complement the neuroanatomical information from VBM to provide data on neural activity (Zuo et al., 2010). ALFF studies have suggested differences between PIGD and TD, yet with inconsistent results. For example, one study linked TD to higher ALFF in the bilateral putamen and cerebellar posterior lobe, but lower ALFF in the bilateral temporal gyrus and left superior parietal lobule (Chen et al., 2015), while another study linked TD to higher activity in temporal lobes, but lower activity in frontal lobes (Hou et al., 2015). Again, this conflicting literature based primarily on small samples highlights the need for larger studies to elucidate potential differences in spontaneous neural activity between PIGD and TD subtypes.

Therefore, we used VBM and ALFF analysis to compare neuroanatomy and local neural activity between reasonably large samples of PIGD and TD patients. We also complemented this neuroanatomical comparison with seed-to-voxel analysis to explore differences in functional connectivity.

## Methods

### Subjects

This prospective study was approved by the Ethics Committee of Henan Provincial People’s Hospital, and written informed consent was obtained from all participants at the Department of Neurology of Henan Provincial People’s Hospital (Zhengzhou, China) between February 2019 and January 2020. The inclusion criteria were as follows: (1) clinically established PD, based on the Movement Disorder Society Clinical Diagnostic Criteria for PD (Postuma et al., 2015), (2) no family history of PD in first-degree relatives, (3) no MRI evidence of structural lesions related to other neurological disorders, (4) no head movement artifacts during the MRI session, (5) no serious cognitive impairment that might affect questionnaire responses, (6) no history of nervous system surgery, and (7) no severe psychosis or psychological diseases.

### Clinical assessment

Patients underwent clinical assessments and MRI examinations while on medication. Clinical data were collected on name, age, sex, disease duration, and medication status. All patients were assessed using the Unified Parkinson’s Disease Rating Scale (UPDRS) (Jankovic et al., 1990) and the Movement Disorder Society’s Unified Parkinson’s Disease Rating Scale (MDS-UPDRS) (Goetz et al., 2008).

Medications taken by the patients at the time of the study were calculated as the levodopa equivalent daily dose (LEDD) (Tomlinson et al., 2010). Disease severity was assessed in terms of Hoehn and Yahr (H-Y) stage (Hoehn and Yahr, 1967) and Part III of the MDS-UPDRS (Goetz et al., 2008). Cognitive function was assessed using the Mini-Mental State Examination (MMSE) after adjusting for age and education level (Crum et al., 1993). Anxiety and depression were assessed using the Hamilton anxiety scale (HAMA) (Hamilton, 1959) and Hamilton depression scale (HAMD) (Hamilton, 1960).

### Assignment to postural instability/gait difficulty or tremor-dominant subtypes

The UPDRS items for TD and PIGD subtypes were used to calculate mean TD and PIGD scores (Jankovic et al., 1990). TD patients were defined to be those whose ratio of the mean UPDRS tremor score (8 items) to mean UPDRS PIGD score (5 items) was ≥ 1.5. PIGD patients were defined as those whose ratio ≤ 1. Patients whose ratios fell outside these ranges were classified as “indeterminate.” In addition, patients who had a positive mean value in the numerator but a zero in the denominator were classified as TD, while patients with a zero in the numerator and a positive mean in the denominator were classified as PIGD. Patients with zeros in both the numerator and denominator were classified as indeterminate.

### MRI

#### Structural MRI

Images were acquired using a 3-T Siemens MAGNETOM Prisma MRI scanner with a 64-channel head coil. The parameters for T1-weighted sequences were as follows: 3-dimensional magnetization-prepared rapid gradient-echo (3D-MPRAGE) sequence, echo time (TE) = 3.43 ms, repetition time (TR) = 5,000 ms, inversion time (TI) = 755 ms, flip angle = 4°, slice thickness = 1.00 mm, number of slices = 208, bandwidth = 240 Hz/pixel, matrix = 256 × 256, field of view (FOV) = 256 × 256 mm^2^, and voxel size = 1.0 × 1.0 × 1.0 mm^3^.

MRI scans were visually checked to exclude those with severe vascular lesions, space-occupying lesions, or motion artifacts. Statistical Parametric Mapping version 12b (SPM12b)^1^ was used to preprocess images and analyze VBM data. First, unified segmentation was applied to the structural T1-weighted images, then the resulting probability maps for gray matter and white matter were spatially normalized to the Montreal Neurological Institute (MNI) template using a high-level non-linear warping algorithm involving diffeomorphic anatomical registration through exponentiated Lie algebra (DARTEL) (Ashburner, 2007). The modulated volumes were smoothed using a Gaussian kernel featuring a full width at half-maximum of 8 mm.

#### Resting-state fMRI

Participants were asked to lie still, relax, and keep their eyes open throughout the scanning session. Functional images were obtained using axial echo-planar imaging with the following parameters: *TR* = 2,000 ms, *TE* = 35 ms, flip angle = 80°, FOV = 240 × 240 mm^2^, matrix size = 94 × 94, voxel dimensions 2.20 × 2.20 × 2.20 mm^3^, slice thickness = 2.2 mm, number of slices = 75, and number of time points = 180.

SPM12b and the CONN functional connectivity toolbox version 18_b (Whitfield-Gabrieli and Nieto-Castanon, 2012)^2^ were used to preprocess images and analyze resting-state fMRI data. Preprocessing of data from all functional sequences involved the following steps: (1) functional slice-timing correction, (2) functional realignment and unwarping (subject motion estimation and correction), (3) functional outlier detection using on-line tools at www.nitrc.org/projects/artifact_detect, (4) structural centering to (0, 0, 0) via translation, (5) functional direct normalization to MNI space, and (6) functional smoothing via spatial convolution with a Gaussian kernel. Subjects were excluded if their head motion exceeded 2 mm in displacement or 2° in rotation. Functional images were resliced at a resolution of 2 × 2 × 2 mm^3^ and smoothed using a Gaussian kernel featuring a full width at half-maximum of 8 mm. Next, regression was used to minimize effects due to head motion or to the presence of white matter or cerebrospinal fluid. Effects due to low-frequency drift or high-frequency physiological noise were minimized using bandpass filtering, such that only frequencies higher than 0.01 and smaller than 0.08 Hz were retained. Systematic shifts were minimized using detrending.

### Statistical analysis

#### Analyses of clinicodemographic characteristics

Statistical analyses of clinicodemographic characteristics were performed using the Statistical Package for Social Sciences (SPSS) for Windows (version 22.0; SPSS, Chicago, IL, United States). Differences associated with *p* < 0.05 were considered significant. Continuous data were reported as mean and standard deviation (SD), while categorical data were reported as frequency and percentage. Differences were assessed for significance using the *t*-test and χ^2^ test as appropriate.

#### Analyses of voxel-based morphometry and amplitudes of low-frequency fluctuation

We performed first-level analysis in the CONN pipeline to generate ALFF maps. To standardize data across subjects, the ALFF of each voxel was divided by the global mean ALFF value using the DPABI toolbox (version 4.0) (Yan et al., 2016).

Smoothed GMV images or standardized ALFF maps were compared between PIGD and TD patients using SPM12 and a significance threshold of uncorrected *p* = 0.001 at the initial voxel level and a false discovery rate-adjusted *p* = 0.05 at the cluster level. Spearson’s correlation analysis and partial correlation analysis were performed to explore potential relationships between neuroimaging findings and clinical characteristics.

#### Functional connectivity analysis

We used first-level analysis of the CONN pipeline to generate functional connectivity maps between the seed and every other voxel in the brain. Correlations were calculated using a general linear model and bivariate correlation weighted by the hemodynamic response function. Differences in seed-to-voxel connectivity between PIGD and TD groups were assessed for significance using a two-samples *t*-test in second-level analysis of the CONN pipeline. The significance threshold was defined as a significance threshold of uncorrected *p* = 0.001 at the initial voxel level and a false discovery rate-adjusted *p* = 0.05 at the cluster level.

## Results

### Clinicodemographic features

Of the 176 patients considered for enrollment, 38 patients were excluded because they were indeterminate. The remaining patients were assigned to PIGD (*n* = 87) or TD (*n* = 51). The two groups did not differ significantly in age, sex, or disease duration (all p > 0.05; Table 1). The PIGD group showed significantly higher H-Y stage, LEDDs and scores on the MDS-UPDRS Parts I-III, HAMD and HAMA (all p < 0.05). Conversely, the PIGD group showed lower MMSE score, although the difference did not achieve significance (*p* = 0.071).

**TABLE 1:** Clinicodemographic characteristics of Parkinson’s disease patients.

Characteristic	TD group (*n* = 51)	PIGD group (*n* = 87)	t/χ^2^ value	*p*-value
Male	31(60.8)	54(62.1)	0.022	0.881
Age at MRI scan, year	61.7 ± 7.5	62.4 ± 6.6	−0.628	0.531
PD duration, year	6.1 ± 3.8	7.1 ± 4.7	−1.308	0.193
H-Y stage	2.2 ± 0.6	2.7 ± 0.9	−4.051	0.001
**MDS-UPDRS scores**				
Part I	9.2 ± 5.2	12.7 ± 6.3	−3.332	0.001
Part II	13.7 ± 6.0	20.3 ± 8.7	−4.783	0.001
Part III	34.7 ± 13.9	42.7 ± 18.9	−2.657	0.009
MMSE score	26.3 ± 3.4	25.1 ± 3.9	1.822	0.071
HAMD score	9.6 ± 4.8	12.3 ± 6.1	−2.958	0.004
HAMA score	9.9 ± 5.8	12.1 ± 6.9	−1.997	0.048
LEDD (mg)	349.0 ± 259.5	527.7 ± 348.4	−3.430	0.001

*Values are n (%) or mean ± SD, unless otherwise indicated. BPV, brain parenchyma volume; GMV, gray matter volume; HAMA, Hamilton Anxiety Scale; HAMD, Hamilton Depression Scale; H-Y, Hoehn and Yahr; LEDD, levodopa equivalent daily dose; MDS-UPDRS, Movement Disorder Society Unified Parkinson’s Disease Rating Scale; PD, Parkinson’s disease; PIGD, postural instability/gait difficulty; TD, tremor-dominant.*

### Comparison of gray matter volume by brain region

**FIGURE 1:**
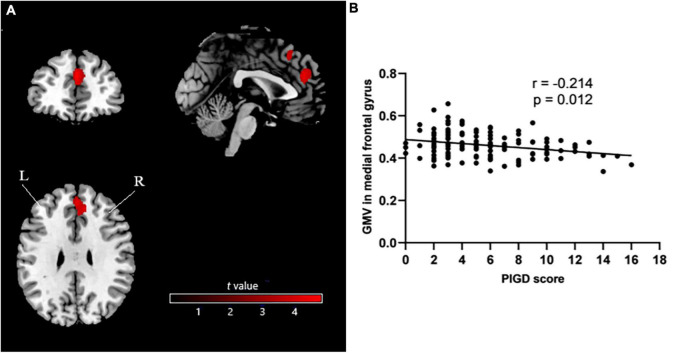
**(A)** Voxel-based morphometry showing smaller GMV in medial frontal gyrus (mainly on the right side) in PIGD patients than in TD patients. **(B)** Analysis of potential correlation between GMV in medial frontal gyrus and PIGD score. GMV, gray matter volume; PIGD, postural instability/gait difficulty; TD, tremor-dominant; L, left; R, right.

In GMV comparisons, age, sex, and disease duration were not treated as covariates because they did not differ significantly between the TD and PIGD groups. The PIGD group showed significantly smaller GMV in the medial frontal gyrus than the TD group, mainly on the right side [MNI coordinates (1.5 43.5 25.5); cluster size, 967 voxels; peak *t*-value, 4.5408, *p* < 0.001 at the voxel level and a false discovery rate-adjusted p < 0.05 at the cluster level; Figure 1A). Among all patients in the study, GMV in the medial frontal gyrus did not correlate significantly with age, disease duration, MDS-UPDRS Part III score, falling score, or freezing score (all p > 0.05; Table 2). In contrast, it did correlate negatively with walking score, gait score, postural stability score, and PIGD score, even after controlling for age (all p < 0.05; Table 2 and Figure 1B).

**TABLE 2:** Analysis of correlations between gray matter volume in medial frontal gyrus and clinical characteristics of patients with Parkinson’s disease.

Characteristic	Uncorrected		Corrected for age	
	r	p	r	p
Age	−0.147	0.085	–	–
Disease duration	−0.049	0.566	−0.071	0.407
MDS-UPDRS Part III score	−0.128	0.136	−0.137	0.111
Falling score	0.127	0.138	−0.159	0.064
Freezing score	−0.093	0.276	−0.153	0.074
Walking score	−0.243	0.004	−0.228	0.007
Gait score	−0.284	0.001	−0.258	0.002
Postural stability score	−0.284	0.001	−0.195	0.023
PIGD score	−0.214	0.012	−0.250	0.003

*MDS-UPDRS, Movement Disorder Society Unified Parkinson’s Disease Rating Scale; PIGD, postural instability/gait difficulty.*

In contrast to the results obtained with all patients, GMV in the medial frontal gyrus did not correlate significantly with PIGD score after controlling for age in separate analyses involving only TD patients (*r* = 0.184, *p* = 0.202) or PIGD patients (*r* = −0.157, *p* = 0.150).

### Comparison of spontaneous brain activity by region

No significant ALFF differences were identified between the PIGD and TD groups in any brain region (*p* < 0.001 at the voxel level and a false discovery rate-adjusted *p* < 0.05 at the cluster level).

### Comparison of functional connectivity

**FIGURE 2:**
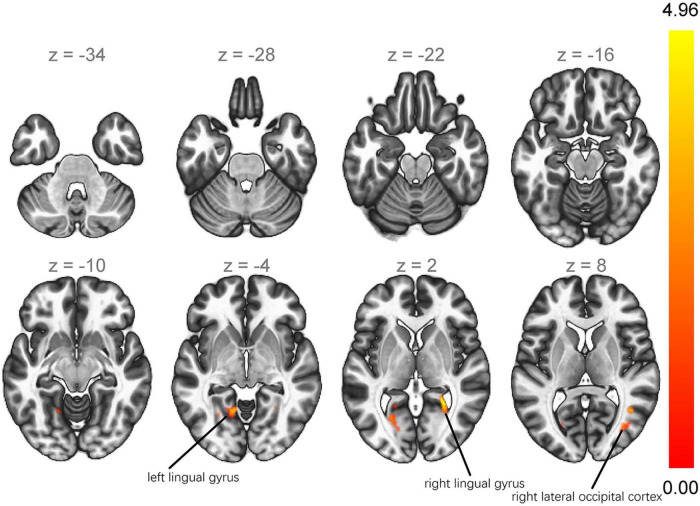
Seed-to-voxel analysis showing greater functional connectivity of the right superior frontal gyrus with the left lingual gyrus, right lateral occipital cortex, and right lingual gyrus in PIGD patients than in TD patients. PIGD, postural instability/gait difficulty; TD, tremor-dominant.

VBM detected neuroanatomical differences between the two groups in the medial frontal gyrus, which was located within the superior frontal gyrus, so we selected the left and right superior frontal gyri as seeds. Seed-to-voxel analysis revealed greater functional connectivity of the right superior frontal gyrus with the left lingual gyrus, right lateral occipital cortex, and right lingual gyrus in the PIGD group (p < 0.001 at the voxel level and a false discovery rate-adjusted p < 0.05 at the cluster level; Table 3 and Figure 2). Among all patients in the study, PIGD score did not correlate significantly with how strongly the right superior frontal gyrus showed functional connectivity with the left lingual gyrus (*r* = −0.010, *p* = 0.931), right lingual gyrus (*r* = −0.035, *p* = 0.747), or right lateral occipital cortex (*r* = −0.003, *p* = 0.978) after controlling for age. The same analysis revealed no significant differences between PIGD and TD patients in connectivity between the left superior frontal gyrus and any other voxel in the brain (p < 0.001 at the voxel level and a false discovery rate-adjusted p < 0.05 at the cluster level).

**TABLE 3:** Seed-to-voxel analysis showing greater functional connectivity in PIGD patients than in TD patients.

Region	R/L	Cluster size		MNI coordinates (x, y, z)		T score	p-FDR
Lingual gyrus	L	336	−12	−56	−2	0.003	0.003
Lateral occipital cortex	R	256	+30	−76	+12	0.007	0.007
Lingual gyrus	R	169	+26	−48	+2	0.029	0.029

*The seed was the right superior frontal gyrus.*

*PIGD, postural instability/gait difficulty; TD, tremor-dominant; MNI, Montreal Neurological Institute; FDR, false discovery rate; L, left; R, right.*

## Discussion

Our study suggests for the first time that patients with the PIGD subtype of PD have significantly smaller GMV in the medial frontal gyrus than patients with the TD subtype. In fact, GMV in the medial frontal gyrus negatively correlated with severity of PIGD symptoms across our entire sample, even after controlling for age. We further found that PIGD patients had greater functional connectivity of the right superior frontal gyrus with the left lingual gyrus, right lateral occipital cortex, and right lingual gyrus than TD patients. Nevertheless, the two groups did not differ significantly in ALFF. Our results identify some neuroanatomical and connectivity differences that may help to explain the differences in presentation, course and prognosis between PIGD and TD subtypes of PD.

Our finding of smaller GMV in the medial frontal gyrus in PIGD is consistent with previous work suggesting greater Lewy body formation and amyloid plaque load in the cortex of patients with PIGD (Selikhova et al., 2009). This greater cortical pathology may, in fact, help explain the more severe clinical characteristics of PIGD compared to TD (Selikhova et al., 2009; Rosenberg-Katz et al., 2013), including extensive atrophy of frontal lobe, parietal, occipital, temporal, and subcortical areas (Rosenberg-Katz et al., 2013). Indeed, our PIGD patients showed higher H-Y stage, LEDDs and scores on MDS-UPDRS Parts I-III, HAMD, and HAMA than TD patients. Numerous studies have documented that PIGD is associated with more severe motor and non-motor symptoms, faster disease progression, and worse daily life experience than TD (Jankovic et al., 1990; Rajput et al., 2009; Ren et al., 2020a,b).

We found that among all patients in our sample, GMV in the medial frontal gyrus correlated negatively with severity of PIGD symptoms, strengthening support for the link between atrophy in the medial frontal gyrus and the PIGD subtype of PD. However, we failed to detect such a correlation in separate analyses of only PIGD or TD patients, which may reflect the small samples. Our results contrast with two VBM studies that failed to detect differences in brain volume between the PIGD and TD groups (Karunanayaka et al., 2016; Al-Bachari et al., 2017). Nevertheless, our results are likely to be reliable because of the reasonably large sample and our strict correction for multiple comparisons.

Consistent with our analysis, studies have linked abnormal function of the medial frontal gyrus to gait problems in PD. The medial frontal gyrus forms part of the medial prefrontal cortex, which is important for maintaining a flexible, effective walking pattern (Hinton et al., 2019). Impaired activation of the medial frontal area may account for patients’ difficulties in initiating movements (Playford et al., 1992), and the PD-associated gait disturbance known as “freezing of gait” (Gilat et al., 2018) has been linked to a bilateral decrease in perfusion of Brodmann area 11 (Matsui et al., 2005) and to abnormal cerebral blood flow (Imamura et al., 2012). High-frequency repetitive transcranial magnetic stimulation of the medial prefrontal cortex can reduce gait freezing and variability as well as improve UPDRS scores (Dagan et al., 2017). In this way, our results and the literature strongly argue that atrophy of the medial frontal gyrus is related to the PIGD subtype in PD. We failed to find a correlation between gray matter volume of the medial frontal gyrus and freezing score, which may reflect that we analyzed all our patients while they were on medication.

At the same time, we found no ALFF differences between PIGD and TD patients. This suggests that there was no difference in spontaneous brain activity between them during the on-medication phase. Our results contrast with previous work that reported higher or lower activity in certain brain regions of PIGD patients (Chen et al., 2015; Hou et al., 2015). This discrepancy may reflect differences in when MRI was performed: we performed MRI only during the on-medication phase, when symptoms may have been milder than during the off-medication phase. Further studies should examine whether ALFF differs between patients of different subtypes during the off-medication phase.

Our finding that most neuroanatomical differences between PIGD and TD lay in the superior frontal gyrus is consistent with a study that found that improvement of motor symptoms after deep brain stimulation of the subthalamic nucleus was associated with an increase in cortical thickness in the superior frontal region (Muthuraman et al., 2017). We found greater functional connectivity of the right superior frontal gyrus with the left lingual gyrus, right lateral occipital cortex, and right lingual gyrus in the PIGD group than in the TD group. It makes sense that dysfunction in the lingual gyrus, particularly on the right side, may trigger walking or balance problems. The lingual gyrus, located in the occipital cortex (also known as visual association cortex), participates in spatial orientation (Schiltz et al., 1999) and visuospatial information processing (Renier et al., 2010; Collignon et al., 2011). The right hemisphere is much more involved than the left one in processing visual information (de Jong et al., 1999; Woolley et al., 2010), so the right hemisphere influences balance (Suarez et al., 2009) and turning (Davidsdottir et al., 2008). However, PIGD score in our sample was not associated with how strongly the right superior frontal gyrus was functionally connected to the left lingual gyrus, right lingual gyrus or right lateral occipital cortex. We hypothesize that the PIGD score reflects the severity of postural instability/gait difficulty, but not the severity of neural network abnormalities.

The extrastriate body area, located in the lateral occipital cortex, integrates visual, spatial attention, and sensory-motor signals in order to represent the observer’s body (Astafiev et al., 2004). The right extrastriate body area may even increase its activity in this respect in order to compensate for loss of function by the dorsal premotor cortex (van Nuenen et al., 2012). This leads us to hypothesize that the enhanced functional connectivity in PIGD brain may compensate for frontal atrophy in order to mitigate postural instability and gait difficulties.

Our results should be interpreted with caution in light of several limitations. First, a patient’s PD subtype can change as the disease progresses; for example, the subtype of some patients shifts from TD to PIGD after approximately six years (von Coelln et al., 2021). This is less likely to affect our results, however, since the average disease duration in our TD group was 6.1 years. Second, we performed clinical evaluations and MRI during the on-medication phase, when symptoms may be milder. This may help explain why we observed no significant ALFF differences between PIGD and TD patients. Future studies should clarify whether spontaneous brain activity differs between the two subtypes during the off-medication phase. Our study lacked a healthy control group, which should be included in future work. Nevertheless, this did not prevent us from identifying differences between Parkinson’s subtypes, which was the focus of our study. One study reported significantly smaller medial frontal GMV in PD patients without cognitive impairment than in healthy controls (Nishio et al., 2010), so whether this holds true for different PD subtypes remains to be seen.

## Conclusion

Our study provides the first evidence that the PIGD subtype of PD is associated with smaller GMV in the medial frontal gyrus and greater functional connectivity of the right superior frontal gyrus with the left lingual gyrus, right lateral occipital cortex, and right lingual gyrus than the TD subtype. Our results may guide further research into the differences underlying the motor subtypes of PD, which in turn may lead to personalized therapies.

## Data availability statement

The raw data supporting the conclusions of this article will be made available by the authors, without undue reservation.

## Ethics statement

The studies involving human participants were reviewed and approved by the Ethics Committee of Henan Provincial People’s Hospital. The patients/participants provided their written informed consent to participate in this study.

## Author contributions

JZ: conceptualization, data curation, formal analysis, investigation, methodology, visualization, writing—original draft, and writing—review and editing. WS: conceptualization, data curation, formal analysis, methodology, visualization, and writing—review and editing. JM: conceptualization, project administration, supervision, writing—review and editing, and funding acquisition. ZW, ML, and SC: data curation and formal analysis. QC, XS, and QG: data curation and investigation. LD: data curation, formal analysis, and resources. DL: project administration and validation. All authors contributed to the article and approved the submitted version.

## Conflict of interest

The authors declare that the research was conducted in the absence of any commercial or financial relationships that could be construed as a potential conflict of interest.

## Publisher’s note

All claims expressed in this article are solely those of the authors and do not necessarily represent those of their affiliated organizations, or those of the publisher, the editors and the reviewers. Any product that may be evaluated in this article, or claim that may be made by its manufacturer, is not guaranteed or endorsed by the publisher.

## Footnotes

1
www.fil.ion.ucl.ac.uk/spm

2
http://www.nitrc.org/projects/conn
